# Serum uric acid and left ventricular hypertrophy in hypertensive patients in Ado-Ekiti

**DOI:** 10.11604/pamj.2020.36.190.21072

**Published:** 2020-07-16

**Authors:** Oladapo Adedamola Adewuya, Ebenezer Adekunle Ajayi, Rasaaq Ayodele Adebayo, Opeyemi Ezekiel Ojo, Olatunji Bukola Olaoye

**Affiliations:** 1 Cardiology Units, Afe Babalola Multisystem Hospital, Ekiti State University Teaching Hospital, Ado-Ekiti, Ekiti State, Nigeria,; 2 Cardiology Unit, Federal Teaching Hospital Ado-Ekiti, Ekiti State, Nigeria,; 3 Cardiology Unit, Obafemi Awolowo University Teaching Hospital, Ile-Ife, Nigeria

**Keywords:** Serum uric acid, systemic hypertension, predictors, target organ damage, LVMI

## Abstract

**Introduction:**

systemic hypertension is a foremost risk factor for cardiovascular morbidity and mortality. Its actions are manifested on organs like the brain, heart and kidneys. High serum uric acid (SUA) escalates cardiovascular vulnerability in patients with systemic hypertension.

**Methods:**

a cross-sectional study was performed in 271 (178 females, 93 males) patients with systemic hypertension. Two hundred and seventy one healthy age and sex matched non-hypertensive persons obliged as controls. Left ventricular hypertrophy (LVH) was estimated by echocardiography. Blood samples were collected for measuring uric acid levels.

**Results:**

mean SUA was significantly higher among the hypertensive patients (371±125μmol/L) than in the controls (269 ± 101.4μmol/L; p < 0.001), and the prevalence of hyperuricemia was 46.9% among the hypertensives and 11.1% among the controls (P < 0.001). Independent predictors of SUA were class of systemic hypertension, left ventricular mass index (LVMI), body mass index (BMI) and age. However, class of hypertension was the best independent predictor of SUA levels in the multivariate regression model (β = 0.597). Linear regression revealed SUA levels ≥ 430μmols/l as a predictor of stage 2 hypertension (F = 26.620, p = < 0.001). Among the hypertensive patients, LVH was present in 39.3% of those with hyperuricemia and in 28.0% of those with normal SUA levels (p = 0.003).

**Conclusion:**

results indicate serum uric acid is positively correlated with hypertension and a reliable indicator of LVH in study population.

## Introduction

Systemic hypertension (SHT) is a major cause of acute and chronic cardiovascular morbidity and mortality throughout the world and a risk factor for Ischemic Heart Disease (IHD) that is the leading cause of death in the world. It´s devastating complications like stroke and Hypertensive Heart Disease (HHD) are the 3^rd^ and the 10^th^ leading causes of death worldwide respectively [1]. The occurrence of SHT is surging in developing countries and it coexists frequently with certain other cardiovascular disease (CVD) risk factors, but is a significant public health challenge that can be prevented and managed [2]. Target organ damage (TOD) related to hypertension are stroke, ischemic heart disease, left ventricular hypertrophy (LVH) and kidney disease [2]. In assessing a patient with systemic hypertension, it is important that TOD be identified in order to improve the individual´s global cardiovascular risk for initiating treatment decisions, and to define target BP levels [3]. In underdeveloped areas of the world, the diagnosis of hypertension is often deferred and, as a result, TOD may be existent at the stage of diagnosis [4]. The Framingham and other epidemiological studies and experimental surveys have shown that hyperuricemia (HU) substantially increases the risk for target organ damage in individuals with systemic hypertension [5-7]. Therefore, measurement of serum uric acid is recommended as part of the screening of patients with systemic hypertension [8]. This study sought to determine the relationship of serum uric acid levels with systemic hypertension and left ventricular hypertrophy.

## Methods

**Study design:** this was a cross-sectional study carried out in the cardiology outpatient clinics of the Ekiti State University Teaching Hospital, Ado-Ekiti, Ekiti State, Nigeria.

**Study population:** a total of 542 participants, 271 hypertensive cases selected by systematic random sampling attending the cardiology clinic and equal number of age and sex matched non hypertensive hospital staff served as controls. Inclusion criteria were all men and women aged 18 years and above with a diagnosis of hypertension by blood pressures ≥ 140/90mmHg and on antihypertensive medications and patients in different hypertensive ranges according to JNC-VII classification [9]. Excluded from the study were participants with respiratory diseases like tuberculosis, on pyrazinamide, asthmatics and chronic obstructive pulmonary diseases (COPD), renal disease, alcoholics, postmenopausal women, patients on uricosuric drugs that is either primary such as benzbromarone, allopurinol, sulfinpyrazone, probenecid and colchicine [10, 11] or secondary uricosuric drugs such as calcium channel blockers (CCBs) like amlodipine, angiotensin receptor blockers (ARBs) like losartan, and atorvastatin, fenofibrate, adrenocorticotrophic hormone and cortisone [12].

The STEPS approach for cross sectional studies was used that included: Step 1: collection of demographic data, Step 2 was the physical measurement of the height, weight, waist, hips and blood pressures. Step 3 was biochemical measurements, which included the collection of blood samples [13] for SUA and cardiovascular risk factor analyses. EKSUTH Ethics and Research Committee approval was obtained before the commencement of the study and informed Consent was obtained. Sources of Information: a. Case Report Forms (CRF): Each participant had a case report form containing information on demographics, various cardiovascular risk factors and anthropometric measurements, duration and class of hypertension, serum uric acid levels, obesity (BMI>30kg/m^2^), and echocardiogram data. b. Uric Acid Assay Method: Uric Acid levels were determined by the colorimetric method using available uric acid assay kit. In this study, Serum uric acid (SUA) is defined as elevated or low with concentrations of ≥ 430μmols/l in men and ≥ 360umol/l in women or ≤ 200 in men and ≤ 140μmol/l in women respectively [14]. c. Blood Pressure Determination: Blood Pressures were assessed with a standard mercury sphygmomanometer by Accoson. SBP and DBP was taken at Korotkoff phases 1 and 5 respectively, to the nearest 2 mmHg [15]. d. Echocardiography: Left Ventricular Hypertrophy (LVH): LVH was assessed with an ultrasound machine type “Sonoscape SSI-1000” equipped with a 2-5Hz cardiac transducer. Patients were placed in the left lateral position and targeted echocardiographic measurements taken i. IVSTd=Interventricular septal thickness in diastole ii. PWTd = Posterior wall thickness in diastole, iii. LVIDd-Left Ventricular Internal Dimension at diastole --just beyond the tips of the mitral valve leaflets. The left Ventricular mass (in grams) was computed using the ASE Standard and indexed to the Body Surface Area (BSA):

BSA (m^2^) = √ [BW (kg) x Ht (cm)/3600] LVM = 0.80 (1.04 (LVIDd + PWTd + IVSTd) 3-(LVIDD) 3) g+0.6g and LVMI = *LVM/BSA* [16].

Relative wall thickness (RWT) was determined in diastole as [17], *2χPWT LVDD* i.e. 2x posterior wall thickness /LVdiastolic diameter. A partition value of 0.45 for RWT was used for both men and women. Left ventricular mass index and RWT were used to define left ventricular geometry. Concentric Hypertrophy was defined as increased Left Ventricular Mass Index (LVMI) with increased RWT while Eccentric Hypertrophy was defined as increased LVMI with Normal RWT and Concentric Remodelling was defined as Normal LVMI with increased RWT. Target organ Damage was noted as LV hypertrophy, defined as LVMI ≥ 134g/m^2^ in men and ≥ 110g/m^2^ in women as above [18].

**Statistical analysis:** data were analysed with the SPSS version 23 software (Chicago, IL, USA), and presented using descriptive statistics such as bar charts, graphs, and tables. Standard Deviation and means was used in presenting continuous variables while discrete and categorical variables were expressed as proportions and percentages. Comparison of means between groups was performed using independent t-test. Correlations between continuous variables like Uric Acid concentrations, blood pressure classes and LVMI were computed with Pearsons and Spearmans correlation coefficients. Multiple regression analysis was used to predict the dependent variable (Uric acid levels) Y, by multiple independent variables, while linear regression was used to predict the value of the dependent variable SUA by the single independent variable, systemic hypertension. The level of significance was set at p < 0.05.

## Results

The mean age of the cases was 60.8 ± 12.3 years. There were 92 males (33.9%) and 179 females (66.1%). Major proportions of the cases (241; 88.9%) were married and had tertiary education (141; 52.0%). Close to half of the cases were retired civil servants (125; 46.1%) and artisans were few. (28; 10.3%). The controls were of similar sociodemographic characteristics. There was no statistically significant difference in the mean ages of the cases and controls (P = 0.811). The waist to hip ratio of the cases (0.92 ± 0.07) were higher than that of the controls (0.81 ± 0.06) and the mean body mass index of the cases was 28 ± 4.9 kg/m^2^, which was significantly higher than that of the controls 25 ±4.4 kg/m^2^; P = < 0.001. The baseline Clinical and laboratory characteristics of the study population is shown in Table 1.

**Table 1:** clinical and laboratory characteristics of the study population

Variable	Cases (n=271)	Controls (n=271)	p-value
Age (years)	60.8±12.6	60.5±14.0	0.811
WHR	0.92±0.07	0.81±0.06	<0.001*
BMI (kg/m^2^)	28±4.9	25±4.4	<0.001*
SBP (mmHg)	161.2±16.1	122±9.7	<0.001*
DBP (mmHg)	103±12.4	83.0±8.3	<0.001*
SUA (µmol/L)	371±125	269±101.4	<0.001*
Hyperuricemia (%)	46.9	11.1	<0.001*
LVMI (g/m^2^)	135.2±53.4	93.4±22.9	<0.001*
LVH prevalence (%)	66.4	19.4	<0.001*
TC (mmol/L)	4.91±0.89	4.78±0.56	0.061
TG (mmol/L)	1.34±0.53	1.24±0.48	0.230
HDL-c (mmol/L)	1.32±0.45	1.40±0.40	0.252
LDL-c (mmol/L)	3.20±0.89	2.81±0.61	0.021*

Notes: data are expressed as mean ±standard deviation, *statistically significant. Abbreviations: n, number; WHR, waist-to-hip ratio, BMI, body mass index; SBP, systolic blood pressure; DBP, diastolic blood pressure; TC, total cholesterol; TG, triglycerides; HDL-c. high density lipoprotein cholesterol; LDL-c, low density lipoprotein cholesterol; SUA, serum uric acid; LVMI, left ventricular mass index; LVH, left ventricular hypertrophy

**Figure 1:**
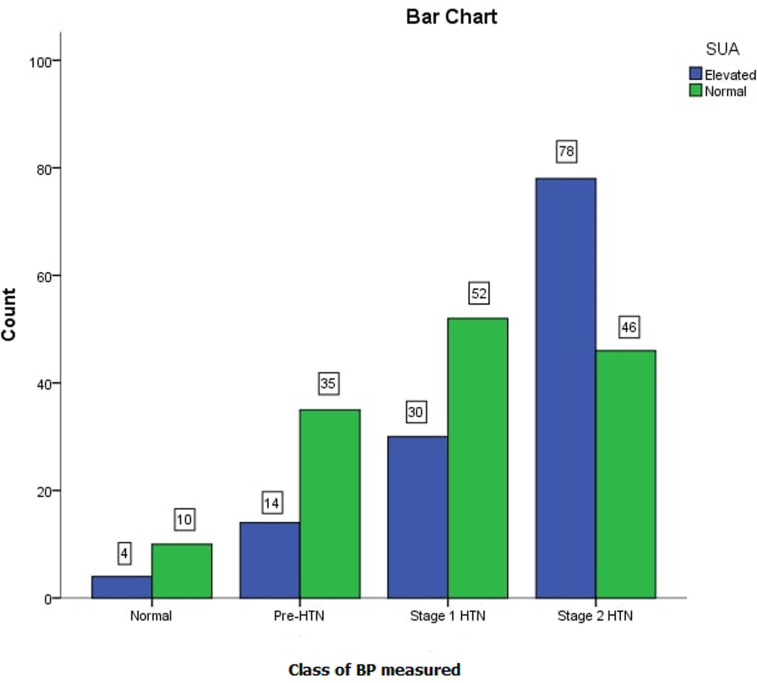
bar chart of elevated or normal SUA levels with stages of systemic hypertension

**Figure 2:**
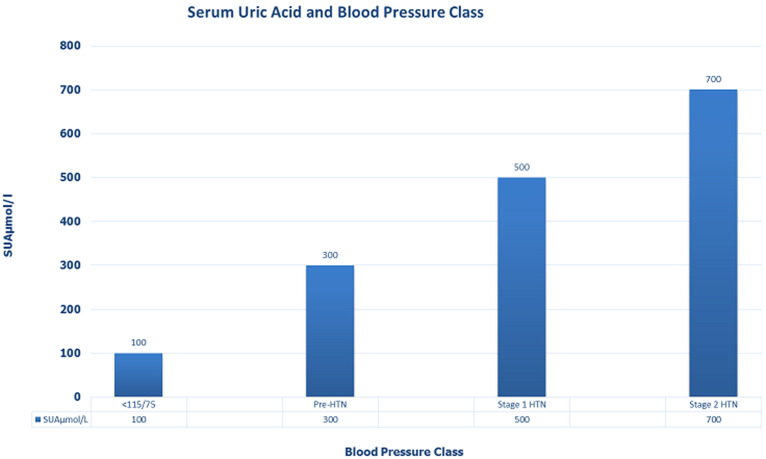
graph of SUA and blood pressure (BP) class

**SUA and correlations with systemic hypertension:** the mean SUA was significantly higher in the cases (371 ± 125μmol/L) than in the controls (269 ± 101.4μmol/L; p<0.001) as shown in Figure 1, more patients in stage 2 hypertension had elevated SUA than those with normal SUA. Elevated SUA correlated positively with different stages of systemic hypertension among the cases (r = 0.325, p < 0.001, Table 2 and Figure 2), suggesting that mean SUA increased with the severity of systemic hypertension (Table 3). Using serum uric acid concentrations (SUAμmol/l) as a dependent outcome, independent predictors of SUA were class of systemic hypertension, left ventricular mass index (LVMI), body mass index (BMI) and age. However, class of systemic hypertension was the best independent predictor of SUA levels in the regression model with β coefficient of 0.597. Linear regression revealed that class of blood pressure is a possible predictor of SUA levels (F=26.620, p=<0.001) (Table 4). Table 4 above provides the *R*and *R^2^* values. The *R*value 0.301 indicates a positive correlation. The *R^2^*value indicates how much of the total variation in the dependent variable SUA, can be explained by the independent variable; class of BP which is 9.1%. Hence class of BP contributes statistically significantly to the linear regression model and predicts SUA levels.

**Table 2:** correlation coefficient of elevated SUA with different stages of systemic hypertension

		Value	Asymptotic Standardized Error a	Approximate Tb	Approximate Significance
Ordinal	Spearman Correlation	0.325 fair relationship	0.057	5.610	<0.001
N of Valid Cases		271			

a. Not assuming the null hypothesis. b. Using the asymptotic standard error assuming the null hypothesis. c. Based on normal approximation.

**Table 3:** multivariate regression analysis for predictors of serum uric acid levels

Variables	Z-score	S.E	Partial r	R2	p-value
Dependent variable: SUAµmol/l					
Class of systemic hypertension	0.597	0.119	0.301	0.491	<0.001
Left Ventricular mas index (LVMIg/m^2^)	0.382	0.113	0.385	0.278	<0.001
Body mass index (BMI kg/m^2^)	0.269	0.117	0.358	0.235	0.002
Age (in years)	-0.015	0.110	0.459	0.021	0.020

Z-score=standardized Beta (β) coefficients r=correlation coefficient R2=coefficient of determination S.E=Standard error

**Table 4:** linear regression analysis for predictor of serum uric acid levels

Model	Standardized Coefficients (βeta/R)	Regression (F)	R Square (R2)	Significance (p)
(Constant) Class of BP Measured	0.301	26.620	0.091	<0.001

a. Predictors (constant), Class of BP measured b. Dependent Variable; SUAµmol/l

**Figure 3:**
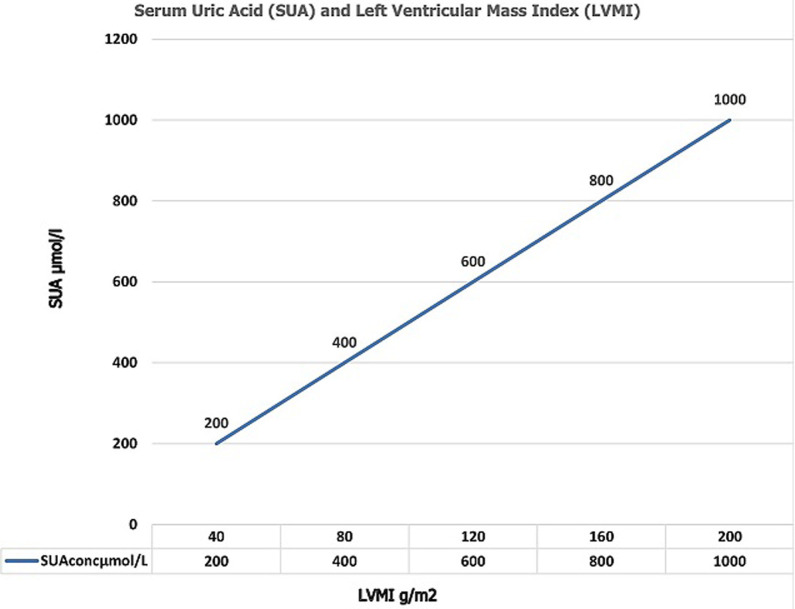
graph of SUA and Left Ventricular Mass Index (LVMI)

**SUA and correlation with Left Ventricular Hypertrophy (LVH):** LVH was present in 66.4% of the cases and in 19.4% of the controls (p < 0.001) as shown in Table 1. Similarly, LVH was more common among the hypertensive cases with abnormal SUA, compared with the hypertensive cases with normal serum uric acid levels (39.3% versus 28% respectively, p = 0.003) as shown in Table 5. As shown in Table 1 and Table 5, left ventricular mass index (LVMI) was found to be significantly higher in the hypertensive cases than the controls, and in the hypertensive hyperuricemic cases than the nonhyperuricemic cases respectively (p = < 0.001). LVMI (β = 0.382; p = < 0.001), was also found to be a predictor of SUA levels in multiple regression analysis, though weaker than HTN (β = 0.597; p = < 0.001), as shown in Table 3. 27.5% was the variance observed in LVMI group. The BMI was also found to be a predictor, but much weaker, of SUA levels (β = 0.269; p = 0.002) (Figure 3). Presence of confounding variables revealed a stronger correlation between the SUA and LVMI at 0.334 vs 0.221 in the absence of confounders, meaning other factors rather than SUA may be responsible for LVMI, as shown in Table 6. Further analysis revealed that low density lipoprotein cholesterol (LDL-c) was significantly higher in the hypertensive cases (p = 0.021), than the controls as shown in Table 1, but insignificant between hyperuricemic cases and non hyperuricemics (p = 0.314) as in Table 5.

**Table 5:** clinical and laboratory specifics of the 271 hypertensive cases with elevated and normal SUA levels

Variable	Elevated SUA mean (SD)	Normal mean (SD)	p-value
**Age (years)**	60.14 (10.8)	61.28 (13.5)	0.450
**BMI (kg/m^2^)**	30.8 (5.6)	28.0 (4.1)	<0.001*
**WHR**	0.92 (0.2)	0.91 (0.2)	0.192
**SBP (mmHg)**	162.5 (15.3)	157.4 (17.3)	0.080
**DBP (mmHg)**	96.9 (11.2)	95.8 (12.9)	0.895
**SUA (µmol/L)**	448.89 (97.2)	267.82 (91.7)	0.001*
**LVMI (g/m^2^)**	145.4 (51.2)	116.1 (44.0)	0.001*
**LVH prevalence (%)**	39.3 (40.7)	28.0 (20.7)	0.003*
**TC (mmol/L)**	5.25 (0.7)	5.20 (1.2)	0.326
**TG (mmol/L)**	1.4 (0.5)	1.39 (0.4)	0.152
**HDL-c (mmol/L)**	1.25 (0.3)	1.37 (0.2)	0.126
**LDL-c (mmol/L)**	3.26 (0.7)	2.91 (0.7)	0.314

**Table 6:** SUA and LVMI correlations with confounding variables

Confounding Variables	SUAµmol/L	Left Ventricular Mass Index (LVMI)
Age, Sex, Exercise, TC, LDL, TG, BMI, DM, Duration and Class of BP and Correlations	1.000	0.334
Correlations	0.334	1.000
Correlation Significance (2-tailed)	0.001	0.001

## Discussion

The key findings in this study is a predominance of hypertensive patients with elevated serum uric acid (SUA) than normotensive controls. This association between elevated SUA and hypertensives was similar to the findings by Poudel *et al*. who carried out a cross sectional study of hypertensive patients in Nepal and found that elevated SUA was more common in hypertensive patients when compared to the normotensive patients (28.8% versus 13.7% respectively; p < 0.001) [19]. However, this prevalence was less than the 46.9% versus 11.1% rate, (as shown in Table 1) established in the present study. Fasae and Omotoso [20] mentioned that the prevalence of hyperuricemia in hypertensive patients was 36.7%, which is lower than the 55% and 52.9% reported by Nagahama [21] and Murugan *et al*. [22] respectively, and higher than figures (26%-33%) reported in studies on Caucasians [23, 24].

In the current study, elevated SUA (> 430 and 360μmol in men and women respectively) in hypertensives had a significant positive correlation with worsening grades of hypertension with the highest proportion of patients with elevated SUA found with Stage 2 hypertension than those with earlier stages of hypertension as shown in Figure 1. In the same vein, reducing levels of uric acid in blood lowered blood pressure to normal in most teens in a study by Feig *et al*. [25]. A number of epidemiologic studies have shown a relation between SUA levels and a wide variety of cardiovascular conditions including hypertension, metabolic syndrome, coronary artery disease, cerebrovascular disease, vascular dementia and kidney disease [25] and Gustafsson mentioned that hyperuricaemia is implicated in the pathophysiology of systemic hypertension, congestive heart failure (CHF), type 2 diabetes mellitus (T2DM), and atherosclerosis, with or without cardiovascular events [26].

The relation between uric acid and systemic hypertension is discerned not only with frank hyperuricemia (defined as more than 6 mg per deciliter [360 μmol per liter] in women and more than 7 mg per deciliter [420 μmol per liter] in men) but also with uric acid levels considered to be in the normal to high range (> 5.2 to 5.5mg per deciliter [310 to 330 μmol per liter]) [27, 28]. It is important to note that, a possible pathogenetic mechanism linking these correlations includes the discharge of free fatty acids from visceral adipose tissue, which escalates hepatic gluconeogenesis. This decreases peripheral tissue glucose uptake, thus causing hyperinsulinemia. Sequentially this causes avid renal salt retention, which increases BP, and urate reabsorption. This process is also associated with the discharge of proinflammatory markers as well as fibrinogen and C-reactive protein that altogether act in concert with dyslipidemia to increase the whole cardiovascular risk of a person [29]. The serum urate level depends on dietary purines, the degradation of endogenous purines, and the renal and intestinal excretion of urate. The dominating factor contributing to hyperuricaemia is under-excretion of urate [30].

In the last decade, several well-grounded pieces of evidence showed that the elevation of uric acid often occurs prior to the development of hypertension or metabolic syndrome, thus suggesting a direct association between elevated SUA and these conditions [31]. Moreover, a recent study by Zheng *et al*. [32], indicated that high serum uric acid concentrations were independently and positively associated with the risk of incident hypertension among the Chinese. Taken together, these observations suggest that elevated SUA may be directly linked to the etiology of high blood pressure. High blood pressure promotes kidney dysfunction which in turn, increases SUA that activates the renin-angiotensin-aldosterone system, promoting cardiomyocyte growth and interstitial fibrosis which are pathologic hallmarks of left ventricular hypertrophy (LVH) [33, 34] This may partly explain the understanding in this study that SUA correlated significantly with left ventricular geometric pattern of the concentric hypertrophic type, as shown in Figure 3 which is consistent with the findings by Viazzi *et al*. [35, 36] where each standard deviation increase in serum uric acid entailed a 75% higher risk of having cardiac hypertrophy of the concentric type. Conversely, their study differs from this one in the sense that they were untreated patients with essential hypertension. The positive correlation in their study remained even after adjustment for body mass index, age, creatinine clearance, and high-density lipoprotein cholesterol. Similarly, Catena *et al*. found that hypertensive patients with LV hypertrophy had higher uric acid levels and a greater prevalence of hyperuricemia than patients with a normal left ventricular mass [37].

In our study, there is a positive linear association between SUA ranges and LVMI (r = 0.221, p = < 0.001) as shown in Table 6. Adjusting for effects of multiple confounders like age, BMI, gender, exercise, lipids, diabetes, duration of hypertension and the class of BP made the correlation stronger (r = 0.334, p = 0.001) meaning other factors apart from SUA affects the development of LVH and its different geometric patterns. On the other hand, Campo *et al*. [38] found that hyperuricemia was not an independent marker of LVH and Tsioufis *et al*. [39] assessing the relationship between SUA and markers of TOD such as LVH and microalbuminuria in 842 nondiabetic hypertensive patients reported that increased SUA levels were associated with microalbuminuria but not with LVH. Our study is similar to Iwashima [40] where he found that SUA correlated with LVMI, and also predicted the risk for future cardiovascular events. In addition, Ganau *et al*. found 17% of the patients studied had concentric LV hypertrophy which is a geometric pattern associated with increased cardiovascular morbidity [41] Comparably, De Scheerder *et al.* [42] also found that SUA correlated significantly with LVMI, and was a unique predictor of the variance of LVmass.

UA is the ultimate breakdown product of dietary or endogenous purines and is generated by xanthine oxidase (XO). A net release of urate in coronary heart disease [42] and the presence of XO in the human heart has been demonstrated [43]. UA may reflect the generation of superoxide and resultant oxidative stress via the XO system [44]. The association between UA and LVMI might relate to an association of UA with other risk factors, especially including renal dysfunction, oxidative stress, severity of hypertension, and obesity. Moreover, the independent association between UA and the severity of hypertension is well accepted [45]. There is also a possibility that UA itself may induce LVH. Preceding reports have shown that UA impaired Nitric oxide (NO) generation and induced endothelial dysfunction and smooth muscle cell proliferation [46, 47] In experimental and in vitro systems, UA appears to have the ability to induce inflammatory mediators, such as tumor necrosis factor α, [48] and potentially stimulates mitogen-activated protein kinase which are known to induce cardiac hypertrophy [49, 50]. These results propose that cardiac hypertrophy may be, at least in part, attributable to an increase in UA itself, via stimulation of endothelial dysfunction, smooth muscle cell proliferation, and inflammation [51]. On the other hand, Brooks *et al*. [52] in a study of 51 University of Michigan professors, mentioned SUA as an endogenous cortical stimulant related to behavioral characteristics (r=0.66) that lead to outstanding performance. This was supported by Mueller *et al*. [53] that indicated that serum uric acid correlated with achievement-oriented behavior. Its effects on various target organs like the brain, heart, kidneys may be related to SUAs (C_5_H_4_N_4_O_3_) similar chemical structure to caffeine (C_8_H_10_N_4_O_2_) that stimulates organs and both have antioxidant properties that are neuroprotective [54] this present study revealed that elevated SUA levels (≥ 430μmols/l) correlated positively with LVMI.

Losartan can cause uricosuria. The Losartan Intervention for Endpoint Reduction (LIFE) study outlined a 29% reduction in composite cardiovascular outcome (myocardial infarction, left ventricular hypertrophy, stroke and cardiovascular deaths) in the losartan (ARB) arm of the study suggesting that a decrease in serum uric acid levels can lead to reduction in adverse cardiovascular outcomes [55] Rekhraj *et al*. elucidated that high-dose allopurinol regresses LVH, reduces LV end-systolic volume, and improves endothelial function in patients with ischemic heart disease (IHD) and LVH [56]. This study also revealed a significantly higher BMI in hypertensive cases than the controls (Table 1), and a higher level in the hypertensive hyperuricemic cases as compared to the hypertensive normouricemic patients as in Table 5. BMI is also a weaker predictor of SUA levels as compared to systemic hypertension and LVMI as shown in Table 3. Mancusi *et al*. mentioned that SUA is positively associated with body mass index (BMI), blood glucose, blood pressure (BP), markers of inflammation, and altered lipid profile but concluded that in treated hypertensive patients, high levels of SUA normalized for major biological determinants and do not independently predict CV outcome [57]. The finding that increased body mass index is associated with increased LV mass index is also consistent with other studies. For example, MacMahon *et al*. reported a reduction of LV mass after weight reduction in young, obese hypertensive subjects [58]. Previous studies have shown that body mass index is a significant correlate of LV mass in both normal children and adults as well as in children and adults with elevated blood pressure [59]. Our study was designed to break grounds on the presence of SUA in hypertensive patients and examine its likely association with left ventricular hypertrophy and other cardiovascular risk factors in our particular environment in Nigeria. To strongly define the causal role of SUA in the incidence of LVH among hypertensive patients, larger studies may be needed.

## Conclusion

This study reveals that hyperuricemia is widespread in our study population with systemic hypertension and both are positively correlated. Hyperuricemia was associated with LVH. Thus, the study recommends a repetitive evaluation of serum UA in all hypertensive patients.

### What is known about this topic

- Elevated serum uric acid (SUA) is a risk factor for Chronic Kidney Disease (CKD);
- Normal levels of SUA is also associated with systemic hypertension;
- Elevated SUA is associated with a high body mass index (BMI).

### What this study adds

- Linear regression revealed SUA levels ≥ 430μmols/l is a predictor of stage 2 hypertension (F = 26.620, p =< 0.001);
- Class of hypertension was the best independent predictor of SUA levels in the multivariate regression model (β = 0.597);
- Among our hypertensive patients, LVH was present in 39.3% of those with hyperuricemia and in 28.0% of those with normal SUA levels (p = 0.003).
